# Health, social and legal supports for migrant agricultural workers in France, Italy, Spain, Germany, Canada, Australia and New Zealand: a scoping review

**DOI:** 10.3389/fpubh.2023.1182816

**Published:** 2023-10-05

**Authors:** C. Susana Caxaj, Eriselda Shkopi, Carmen T. Naranjo, Alexa Chew, Yi Ting Hao, Michelle Nguyen

**Affiliations:** ^1^Athur Labatt and Family School of Nursing, Western University, London, ON, Canada; ^2^Dipartimento di Filosofia e Beni Culturali, Università Ca’Foscari Venezia, Marie Skłodowska-Curie Global Post-Doctoral Fellow, Venice, Italy

**Keywords:** migrant agricultural worker, social support, health care, legal services, occupational health, vulnerability, services, protection

## Abstract

**Introduction:**

We carried out a scoping review to examine what previous literature can teach us about practices and possibilities for support services for migrant agricultural workers.

**Methods:**

Following guidelines for scoping reviews as outlined by Arksey and O’Malley (2005) and further refined by Levac et. al (2010) we conducted searches of several databases and two additional searches to capture regions of focus and more current literature. We used a thematic analysis to generate our themes.

**Results:**

Our analysis yielded four key themes: (1) political, economic and legal factors; (2) living and working conditions; (3) facilitators/barriers to navigating services and supports and; (4) potential and existing strategies for social support for migrant agricultural workers. The first two themes pointed more to structural and material conditions that both posed barriers for this population to access supports, but also illustrated vulnerabilities that pointed to the need for a variety of services and protections. Under the third, we highlighted the ways that the design of services and supports, or their degree of accessibility, could shape the level of help available to this population. Lastly, potential and existing strategies for social support discussed in the literature included an emphasis on mental health and wellbeing, occupational health and safety training and documentation, and policy reforms to secure the status and address the precarity of this workforce.

**Discussion:**

While research on social support and service provision for migrant agricultural workers is still in its infancy, a strength of this body of work is its attention to macro-level issues that advocate for strategies that address root factors that shape this group’s health. Further research is required to expand our understanding of social support roles and possibilities across other domains and sectors for this population.

## 1. Introduction

Health, social and legal services and protections are often lacking, limited, or inaccessible to migrant workers throughout the world (1). For migrants working in agriculture in particular, being located in rural and remote small towns, often residing on employer’s property, and working in one of the most hazardous sectors complicate their ability to seek help and protection (2, 3). Under a broad conceptualization of social support, that encompasses legal, health and social formal services and informal (e.g., volunteer) support (4), we sought to examine what is known in the literature on this topic as it pertains specifically to this workforce. Employing a scoping review framework (5, 6), and a thematic analysis approach, we report here on our analysis of the literature situated in the regions of Canada, Australia, New Zealand, France, Italy, Spain and Germany. Our research question guiding our analysis was: *What can prior literature teach us about social support services and strategies for migrant agricultural workers?*

In this article, we focus on migrant agricultural workers (MAWs) who arrive to a foreign country without permanent residence, authorized and/or currently working in the agricultural sector, and/or required to return to their country of origin after a specific period of time (e.g., 8 months,2 years) (1, 3). We define social support as help provision that encompasses several domains (e.g., health, legal, social, spiritual) carried out by both formal and informal actors. In the case of migrant agricultural workers, the most common formal actors include occupational health clinics, primary care providers, settlement organizations, consular officers/liaisons and legal advocates (including labor representatives). Informal supports can include peers, family members, civil society organizations and parish communities (4). We argue that while temporary migration programs differ as a result of national policies and bilateral agreements across the world, certain structural conditions and barriers hold relevance across national contexts. Therefore, recommendations and interventions for support provision and service delivery likewise may have some potential across national boundaries.

Practitioners working across sectors and disciplines toward a holistic vision of public health may be best equipped to advocate for, and lead, the development of support provision for migrant agricultural workers. Yet in order to address support gaps faced by this population, interventions are required not only at the program level, but also, at the level of national and international policy change. Future implications for practitioners and decision-makers are discussed in light of key research findings identified through our review.

## 2. Background

More and more, higher income countries (HICs) have turned to migrant labor as a solution to solving labor shortages in agriculture. This turn has been facilitated by inequitable outcomes of globalization that have disrupted local economies in lower- and middle-income countries, and neoliberal markets that have pressured the modes of agricultural production (7–9). Labor shortages in agriculture in turn, have been seen as a result of the seasonality of the work offered, and the known risks and insecurities faced by workers in agriculture. Likewise, HICs have sought to manage migration (10). In countries such as Canada, Australia and New Zealand, entry pathways have turned toward temporary circular migration schemes over opportunities for migrants that allow a clear transition to permanent residence (11). In many European countries, most migrants who work in agriculture have entered host countries as “irregular migrants,” or seeking international protection under Geneva Convention, or other forms of humanitarian protection under EU or national laws, limiting their access to basic protections and services, even as new temporary migration schemes across Europe develop. Furthermore, international agencies in Europe and beyond continue to invest in temporary migration schemes as mechanisms to deter, manage and return unwanted migrant populations (12, 13).

Temporary migrant labor programs are characterized by work and migratory permissions that allow workers to stay and work in the host country for a predetermined length of time (14). This period can range from 5 months, 8 months, or 1 to 4 years depending on the region of destination. The work undertaken by workers in such programs is typically categorized as “low-skilled” and entry-level regardless of the years an individual has been participating in a program (15). Labor laws may require that these workers be paid minimum wage, although practices related to piece-rate pay (15, 16) may threaten this entitlement. Temporary migration programs in Canada, the United States, Australia and New Zealand dictate workers’ terms of employment be in relation to a specific employer, a condition that poses significant precarity for this workforce (16). While in the European Union, the Seasonal Workers Directive (17, 18) outlines that migrants are allowed to change employers, common challenges of language barriers, lack of ties with the host community, recruitment debts, occupational health and safety risks and susceptibility to exploitation remain challenges faced across national contexts (19, 20). Even amidst precarity, migrant workers may be motivated to remain in guestworker programs due to limited financial opportunities in their countries of origin, and the wage differentials between host countries and their countries of origin (21, 22). Push and pull factors interplay, on one hand for migrants seeking work outside of a designated program, agricultural work may be one of the few sectors in which they can gain employment, despite the relatively poor compensation in comparison to other industries. On the other hand, this sector’s increasing workforce shortage functions as a pull factor. Scholars have defined these complexities and interconnection as a system of ‘humanitarian exploitation’ to describe how ‘humanitarian government is functional both to the regulation of the migrant workforce and to the maintenance of the industrial agri-food system.’ (23–26).

As a result of the well-documented structural vulnerabilities inherent in such temporary programs, the need for greater oversight and safeguards to address exploitation of temporary migrants has been reiterated in the academic literature (16, 27). HICs have launched a variety of initiatives with the aims of mitigating such concerns. Strategies have included federal and provincial funding to enhance public health protections in response to COVID-19 (28, 29), mechanisms to promote regularization of migrant laborer’s (30) and investments in regulatory oversight and health and social protections (31). While certainly warranted, limited research has explored the degree to which evidence is guiding the particular initiatives that are implemented, and, the potential of such strategies to mitigate the challenges that are often seen with temporary migration programs. Emerging research does underscore the promise of community membership in trust-building (32), anticipating barriers in support provision (4, 33), multi-sectoral partnerships in service delivery (34, 35), and the challenges amidst funding and organizational climates in which service is enacted (36). Yet for the most-part, this body of work remains highly localized, as well as sector and program-specific. At a policy level, regularization or more accessible pathways to permanent status have been identified as a strategy to proactively address structural vulnerabilities that can influence various hardships faced by migrant agricultural workers (10). Yet this work is rarely in conversation with intervention-based scholarship that could incrementally address the precarity faced by this population. Drawing from the international body of literature, our scoping review will contribute to the systematization of knowledge that can inform an evidence-informed approach to support provision for migrant farmworkers. This synthesis of academic literature can provide a starting point for both broad-based policy interventions as well as program-specific planning that can be launched by health and social care providers interested in better supporting migrant farmworkers.

## 3. Procedures

We were guided by Levac et al. (6) and Arksey and O’Malley’s (5) recommendations for conducting a scoping review. This involved following the five steps outlined under this approach: (A) purpose and research question, (B) identifying relevant studies, (C) selecting studies, (D) charting and analyzing the data and (E) reporting the results. Activities under each step are outlined below.

- *Purpose and research question.* We sought to identify scholarly articles that could provide us with insight about social support services and strategies for migrant agricultural workers. We focused on articles written in English or Spanish. Initially, our scope was inclusive of all industrialized nations settings, including G7 countries (narrowing of this design explained below).
- *Identify relevant studies balancing feasibility with breadth.* To balance these two contrasting quality indicators for a scoping review, two members of the research team developed and piloted a relevance scale, to both identify the most relevant databases, and, to ensure consistency of eligibility. Our relevance scale outlined three eligibility domains: (a) findings specific to migrant agricultural workers (and at minimum, inclusive of); (b) authors implementing, discussing or providing recommendations of social support interventions or initiatives; and; (c) carried out in an industrialized nation and/or G7 country. After reviewing the top 50 hits across five databases, we refined our search and refined our relevance scale to capture a greater range of relevant articles depending on the database. Then, searches were run again in the three databases found to be the most relevant (CINAHL, Scopus, Sociology Collection). MeSH terms (where applicable), synonyms and related concepts related to terms such as migrant farmworkers, seasonal farmworkers, migrant agricultural workers, social support, social services, community-based programming, preventative services, policy, health promotion, casework, and intervention, labor organizing and education were included in the search. See Appendix A for the search syntax used for databases. This original search, conducted in April of 2020, yielded 1929 articles, once duplicates were removed this number came down to 1566. Abstract review immediately identified several articles ineligible on the basis of demographics (*n* = 165), and date of publication (*n* = 96). Several articles were also not accessible to the authors (*n* = 40). Following this initial screening, a full-text review of 1,265 articles followed for eligibility. At this point, we opted to refine our geographic area of interest, and to only include articles from Australia, New Zealand, Canada, France, Italy, Spain or Germany. This only had implications for articles that otherwise would have been included from the United States, as no articles from China, Japan or the United Kingdom were considered relevant based on other eligibility criteria (i.e., article not on migrant agricultural workers, not on social support) and articles from other regions of Europe were not retrieved. A total of 1,201 entries were deemed ineligible, on the basis of being outside of the geographic area of interest (*n* = 71), not relevant to migrant agricultural workers (*n* = 434), not applicable to the concept of social support (*n* = 58) or otherwise, not relevant (*n* = 518). A significant number of articles were focused on capturing the prevalence of certain medical conditions (*n* = 76) which was also deemed irrelevant to our scoring review. Non-academic entries were also excluded (*n* = 44). On the basis of limited current literature based in European countries of interest, one co-author carried out an additional search, identifying an additional 31 potentially relevant articles to include from France, Italy, Spain or Germany through citation searching and website review. Following full-text screening of this section, only 5 were found sufficiently relevant, resulting in an original extraction of 69 articles.
  A secondary search was conducted using Google Scholar in January 2023 in order to include current articles from 2021 and 2022. For the sake of feasibility, the first 100 entries were reviewed. From those entries, an additional 2 were included in the initial search based on reference searching of the full-text of these 100 entries. Of these 102 articles screened, 51 were excluded on the basis of being the wrong population of focus, 32 were excluded because they were based in a regional setting outside of the countries of focus (i.e., not Canada, United States, NZ, Spain, Italy, France, Germany), 2 articles were not relevant, and 1 article identified was a duplicate of an article already included in the review. This left an additional 15 articles which were extracted and incorporated into the analysis. See PRISMA reporting table. 
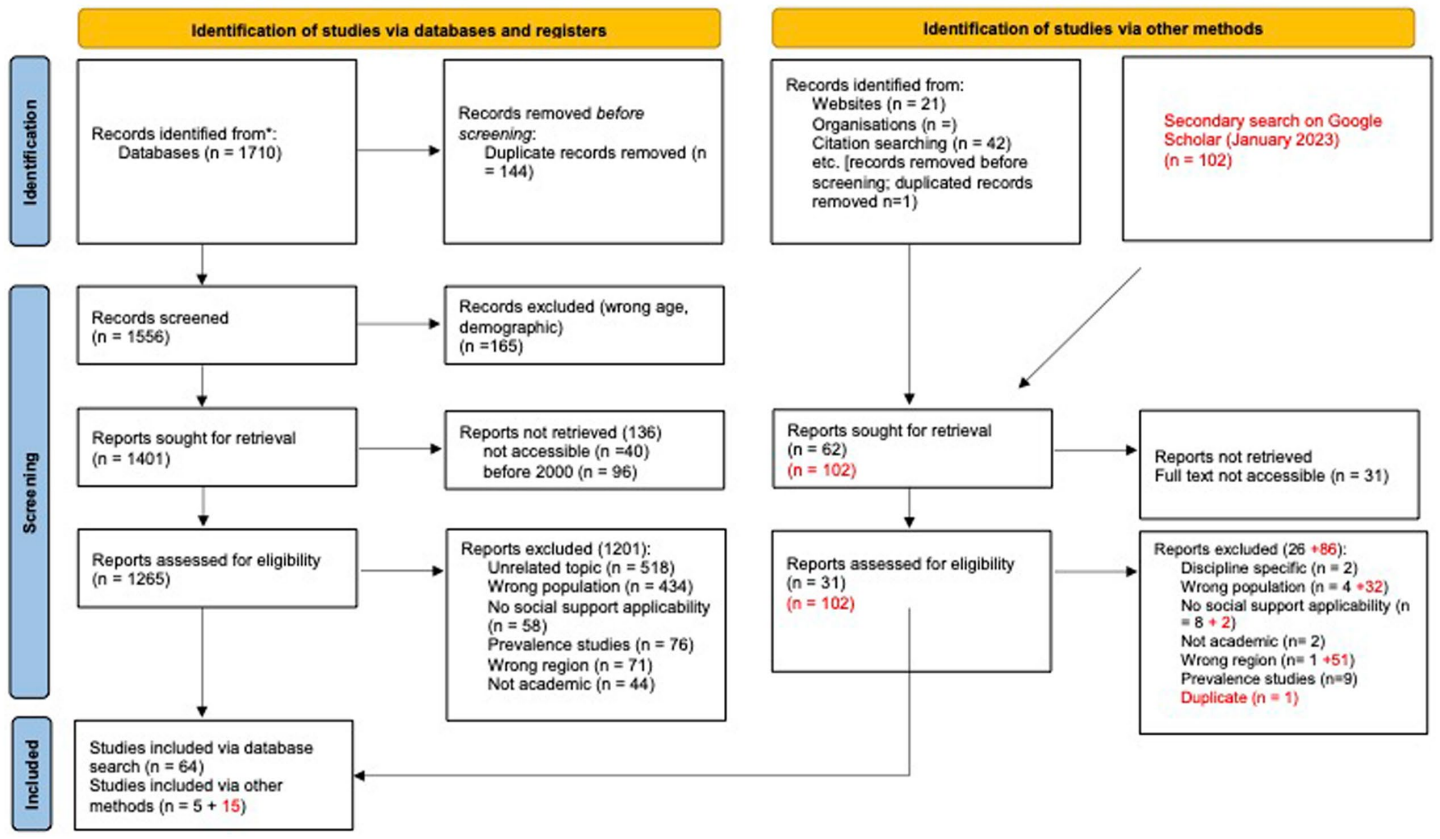
- *Select studies using an iterative team approach*. All articles were imported into a reference management platform for team screening. To screen articles, all team members were provided with training on the use of the relevance tool. To ensure consistency across screening, 20 articles were screened for eligibility, with the team meeting halfway through the process to clarify parameters and criteria for eligibility. Following the joint-screening, further clarity on the relevance scale was developed to ensure consensus across research team members about terms of eligibility of articles screened. Search terms used for the secondary search and the additional search of select European literature is available as Appendix B. The final list of articles included in the analysis is available as Appendix C.
- *Charting the data, qualitative thematic analysis and* (5) *Collating, summarizing and reporting results.* Using a shared online document, all research team members tabulated the region of the study/article, country of origin of participants, methodology, types of interventions or recommendations proposed in regards to support interventions and other data relevant to understanding social support gleaned from the article. Monthly meetings were held to discuss key issues emerging from the screened articles. Using extraction table results, and reference to the original article, research team members coded each extraction unit (article tabulation), populating several fields of an online data-pooling form to better consider the relevance of the article (e.g., research setting, relevance to social support, key findings). The PI collapsed key codes and preliminary themes captured in the online document and presented it to the team. Once consensus on each theme was reached, the team worked together to revisit articles for fit with the emerging thematic framework. All key ideas were found to fit with this framework through several team meetings. Study findings are thus organized across these four domains (see below).

## 4. Findings

Our scoping review indicates that the research on social support for migrant agricultural workers (MAWs) is limited. Yet a close reading of articles that at least hint at social support, provide some insight into migrant agricultural workers’ social support experiences, and further implications for services and strategies in this regard. First, scholars have put emphasis on the structural factors, such as the *political, economic and legal factors* that determine the conditions that migrant agricultural workers face, not only in their destination country, but also, from their position as citizens of countries in the Global South. Second, research has begun to describe the various environmental factors, especially the *everyday living and working conditions*, that are often substandard, as a result of structural conditions, but also, made real as a result of more immediate power struggles, material realities and workplace protocols. While these two categories of research are not intended to explain social support services and strategies for migrant agricultural workers, they do point to specific social supports needed by this population. Furthermore, they help us begin to understand the complex contexts that shape this population’s ability not only to seek, but also to contemplate and assess their social support needs. Third, some research has begun to document specific *facilitators and barriers to navigating services and supports*, including accessibility of existing programs or services. Fourth, and lastly, a few studies have actually introduced and evaluated a specific program, or, provided evidence to inform *future actions to enhance or deliver on social supports* for migrant agricultural workers. Each of these four themes will be discussed in further detail below.

### 4.1. Political, economic and legal factors

Of the regions included in this scoping review, the Canadian and European literature of focus most explicitly identified the role of broader factors in limiting access and entitlements to supports and protections for migrant agricultural workers. Some research for example focused on the limitations and denial of meaningful rights as a result of migrant workers’ temporary status (15, 37). Smith (38) carried out a legal analysis to examine how workers’ constitutional right to freedom of association as agricultural workers in Ontario, Canada had been denied. Others have noted that migrant agricultural workers’ fear of deportation and reprisal, coupled with their limited earning potential, created a coercive dynamic by which they were inclined to accept both unsafe work conditions and limited access to services and protections (2–4, 39–42). Several studies conducted in Canada show how MAWs participating in seasonal programs face several barriers to access rights, health, wellbeing and to construct a sense of belonging (2, 39, 43, 44). Indeed, their long-term presence as circular migrants, did little to demarcate their precarious status as “temporary” migrants’ (4, 45). Moreover, the lack of basic policies to protect this workforce, unreliable sending country official representation, and assumptions of workers’ ineligibility for certain programming (e.g., as “temporary) increase this marginalization (4, 37, 46). Toh and Quinlan’s (42) research in the Australian context generally spoke to the need to investigate and improve visa programs toward addressing the precarious status of temporary migrant workers. Furthermore, despite having recognized legal entitlements this workforce faces several difficulties accessing such things, among them: (a) their ‘outsider’ status, stereotyping and discrimination (including by state agencies); (b) being contracted in precarious labor with little union representatives and; (c) relatedly, participating in industries where complaints to occupational health authorities and workers’ compensation claims are lacking or difficult to file (42).

Speaking specifically of migrant laborers in the Spanish and Italian context, some scholars caution that migration status alone cannot be used to explain the vulnerabilities faced by migrant workers without consideration of economic and social conditions they face (13, 47, 48). Reflected in the work of other scholars, these considerations include the nature of the work afforded to migrants that is typically more hazardous and with limited compensation (40). During the spread of COVID-19 in Italy, Tagliacozzo et al. (19) analyzed the working and living conditions of MAWs, documenting the interplay among structural and systemic vulnerabilities in relation to agricultural, migration and the national health system (NHS). These scholars and others (23) found MAWs face even further complexity as a result of exposure to dangerous and exploitative working conditions that worsened during the pandemic. Nonetheless, migration status in combination with participation in a segmented labor market does contribute to various stressors, whereas years of continuity in a country can play a protective role (49).

Migrant workers may also work under expectations of ‘flexibility’ resulting in replaceability and exploitation and limited policies to protect employment conditions (13, 15, 42, 48, 50, 51). This precarity is worsened by regulatory regimes that place the onus on workers to bear the risk of reporting violations and misconduct, amidst a social climate in which employer surveillance is normalized and enabled (4, 27, 36). Moreover, the categorization of this workforce as ‘essential workers,’ and regulations that showed promise to improve legal status, were revealed to be largely rhetorical and insufficient to address MAWs’ vulnerability to disease. Consequently, their ‘disposability’ went largely unchallenged (15, 52).

Scholars also identify being housed in employer-provided property and limited or inconsistent eligibility for employment insurance across some job categories as factors that can contribute to migrant agricultural workers’ vulnerability. These conditions are exacerbated by a general discourse or expectation that migrant workers be merely workers, and thus, not presenting with social (and health) needs (47, 53). In this same vein, Brickenstein (54) underlines that even though seasonal workers programs are widely advocated as triple wins for: migrants, countries of origin and countries of destination, in actuality, they burden migrants with several challenges that cross-cut at least three domains: (a) limited freedom; (b) limited access to protections/benefits and (c) restricted abilities to form labor unions. Examples include financial risks related to recruitment, stringent visa requirements, limited opportunities to change employers, and contributions to national benefits (e.g., unemployment and retirement fund) without the ability to access them (e.g., Canada, Germany, Australia) (22, 23, 26, 37).

Amidst these structural vulnerabilities, a few studies have considered the role of labor organizing and unions as sources of advocacy and support. While crucial for addressing migrants’ segregation and ‘bridging’ these workforces with the wider community, labor unions have varying approaches to addressing the temporary status of workers, ranging from acceptance to problematizing this reality (37, 42, 55). Yet likewise, labor unions are also vulnerable to the same precarity as placed on migrant workers, limiting the level of support that they can offer migrant agricultural workers (51). In European countries such as France and Germany, formal recognition of the right to unionize, through multiple legal frameworks, exists. Nevertheless, as a consequence of legal and social conditions (e.g., fear of job loss or residence permit) MAW participation may be heavily conditioned (54). In contrast, in Canada, migrant agricultural workers may be subject to certain laws that explicitly exclude them from participation in collective bargaining and prevent adequate protection of their ability to exercise these rights (56, 57).

### 4.2. Living and working conditions

Several studies have illustrated the isolation and segregation from the wider community that is faced by migrant agricultural workers. A sense of alienation is often exacerbated by geographic factors (rurality, remoteness), as well as social experiences of racism, xenophobia and accessibility issues as a result of language barriers (2, 39, 40, 47, 58). A lack of connection to the wider community has drastic consequences for migrant agricultural workers’ ability to pursue their rights, entitlements, and services (2, 13, 37, 58, 59). As a result, migrant agricultural workers are often limited to the mutual support they can provide one another and their families back home (39, 53, 60). Yet this group often faces poor and substandard housing (2, 13, 41, 44, 47, 61) that can result in conflict and competition in housing quarters, rather than cooperation and meaningful support (58, 62). As a result of these poor conditions, migrant workers may also be more susceptible to harms resulting from climate change and climate disasters (13).

Given that MAWs face limited mobility and opportunities to leave their place of work, and that the workforce generally resides on employer-provided property (39, 47) their relationship with their employer often determines this workforces’ access to support (2, 44, 47). Unfortunately, employers are often viewed by migrant agricultural workers as service gatekeepers, and the power dynamic at play between employers and employees can create new barriers for seeking support (13, 47) and reporting abuses by employers (13, 41, 47, 48). For instance, Perry (62) found that workers would censor their concerns and emotions in front of a supervisor, while Preibisch and Otero (3) found that workers would refrain from taking breaks out of concern that it would negatively affect their relationship with their employer. Likewise, Blackman (63) found that farmworkers hid chemical safety concerns from their employers for fear of job loss. Scholars have also found several instances of forced isolation among this workforce, and barriers in reporting health injuries may result in job loss and repatriation (42).

Barriers to adequate health and safety resources and environments have also been noted in literature about migrant agricultural workers. This is particularly relevant for this group as one’s status as a migrant and/or with precarious status, and/or racialized identity and/or working in agriculture have been shown to be correlated with poorer working conditions (44, 64). Kosny and Allen (65) found that migrant agricultural workers in Australia were not properly informed about their roles in injury prevention and workplace safety, being solely responsible for finding resources, and often, not being aware of any. Other research reports limited use of personal protective equipment (PPE) within the farm setting among this population, resulting in likely significant pesticide exposure (66). Colindres et al. (2) found that training was often considered inconsistent or insufficient among MAWs. A potential implication of these gaps in protections may be that there is a need for more safety representatives to address workplace risks and prevent hazardous incidents, especially because prior research indicates that those with safety representatives are more likely to be better protected in the workplace (67). More proactive and regular inspections of workplace health and safety conditions have also been recommended (47, 68, 69).

Translation and interpretation services, as well as careful consideration of cross-cultural communication needs among this workforce, are crucial toward an effective health and safety program in the workplace (67, 70). While industry associations may play a role in promoting migrant workers’ health and safety, prior research suggests that these entities do not view protecting workers’ health or wellbeing as within their mandate (71). Peers, including direct managers may also be important to setting a safety standard in the workplace, as their attitudes and behaviors have been found to have the ability to positively impact workplace safety (72). On the other hand, poor pay, as well as poor working and living conditions make migrant workers more susceptible to developing mental health problems (60, 73). During COVID-19, determinants of both physical and mental health were exacerbated (25). For instance, under-reporting of injuries, social isolation and poor living conditions had significant and even tragic consequences for workers (47, 68). Likewise, poorly and inconsistently implemented public health precautions further contributed to the untimely death of MAWs (44). This research speaks to the need for health care approaches that account for the day-to-day risks and challenges uniquely faced by MAWs.

### 4.3. Facilitators/barriers to navigating services and supports

Language was a significant barrier cited in the literature. Language barriers impacted MAWs’ ability to seek support (2, 13, 41, 42, 60, 63, 74, 75). Along with a lack of formal education and inaccessibility of safety materials, language barriers also created higher risks of occupational injuries (42, 63, 65). Language barriers also presented a challenge for accessing quality healthcare (2, 74, 76–78). For instance, there is often a lack of interpretation or translation services for effective communication between MAWs and health care providers (2, 60, 74, 76–78). Additional barriers to healthcare access include conflicting work hours with health clinic hours, lack of transportation from rural sites, long wait times, lack of childcare services, and precarious status (3, 41, 58, 74, 77–79). Gaps in knowledge of services was cited as another barrier for MAWs in accessing social and health support. Obstacles may include difficulty navigating the health system and a lack of information about available services (78, 80). Colindres, Cohen and Caxaj (2) found a strong disjuncture among MAWs’ intention to report and ‘limited knowledge of basic legal processes’, for instance on how to initiate a complaint or report. Sexual health among MAWs presents another set of unique challenges. Narushima et al. (80) and Wong et al. (79) discussed the gap in knowledge of safe sexual practices, lack of public health initiatives that reached this vulnerable group, and the stigma attached to seeking care for sexual health as factors that dampened the quality of care and likelihood of seeking help among MAWs. Migrant workers also often lack knowledge of legal rights which impedes their awareness of job entitlements (2, 42). This is exacerbated by language barriers and cultural differences which negatively impacts communication between MAW and employers, as well as the larger community they are a part of (60). These factors exacerbate social exclusion and isolation and impede the creation and nurturing of a sense of belonging (2, 37, 39, 41, 47, 61, 65).

There are also some unique challenges in different countries and regions. For example, in New Zealand, health insurers often do not cover treatment for pre-existing health conditions that workers may have prior to entering the labor program. This creates further challenges for MAW receiving proper care (76). Likewise, Peach (78) found that the cost of healthcare, and lack of health insurance were common barriers to healthcare access for this workforce in the Australian context. In Ontario, Canada, where there is universal health care, Wong et al. (79) discussed the difficulty in obtaining OHIP (Ontario Health Insurance Plan) cards for MAWs employers are often responsible for acquiring OHIP cards for MAWs but do not always comply (74). Prior research also shows MAWs often pay out of pocket to access medical services (2). In British Columbia, Canada, Caxaj and Cohen (4) found that working contracts often are not translated into languages that workers can understand, limiting non-English/French speakers’ knowledge of their labor rights. Poor networks, both formal and informal along with a lack of trust in government agencies may also constitute barriers (4).

In the Canadian context, a consistent critique has been the federal governments’ approach to delegating infection control and health access protocols to employers (2, 15, 47, 77). Yet a lack of recognition of the unique vulnerabilities faced by this group, and an off-loading of responsibility to safeguard workers’ rights is a common theme across national contexts. In Italy and Canada alike for instance, researchers have found that healthcare workers often lack basic knowledge on MAWs’ entitlements to health services, as well as MAWs’ migration and work context that impacts their health status (19, 47, 77). Lastly, cultural barriers and isolated geographical locations can impact the quality of support that is accessible or offered to MAWs. Such contexts limit MAWs’ physical access to the larger community they live in and social supports that are offered (13, 44, 58, 74). Due to a lack of public transportation in many regions, workers may also have a reliance on their employers or intermediaries for transportation/service navigation (2, 13, 47, 58, 74).

### 4.4. Potential and existing strategies for social support for migrant agricultural workers

The literature suggests many areas of improvements to better deliver needed supports and services for MAWs. Below, we outline the following themes identified in the literature (a) securing migrant agricultural workers’ status and living conditions (b) promoting mental health and wellbeing and; (c) addressing poor working conditions through occupational health practices and research. Under these headings, we also outline existing supports that are documented in the literature.

#### 4.4.1. Securing migrant agricultural workers’ status

Many vulnerabilities faced by MAWs are related to their status as temporary non-citizens (15, 37, 42, 43, 46, 58, 81). Migratory status has also been shown to influence migrants’ exposure to hazards (19, 42, 49, 61). These realities emphasize the need for further examination of the potential of secure status to address basic legal, health and social challenges often faced by this workforce.

Notably, in recent years, scholars have documented status adjustments during Covid-19 for ‘essential’ workers (MAWs and caregivers) in several countries including Italy, Canada and France. On the one hand, nation-states’ quick willingness to make such adjustments indicate that there is some awareness of how status is vital to accessing basic rights. On the other hand, an examination of such changes also underline that these minor and temporary changes are insufficient to address the structural precarity that often goes hand-in-hand with the design of MAWs labor participation (15, 44, 81). Temporary and precarious status also shape the degree to which MAWs accept substandard health, housing and working conditions, and, the degree to which they are willing to refuse unsafe or hazardous conditions and advocate for their rights (4, 13, 42, 47, 61). As a result, several scholars and advocates worldwide see regularization, permanent residence and other policy reforms that contribute to a more secure status as crucial to mitigating migrant workers’ precarity and marginalization (13, 15, 43, 61). In addition, specific measures to protect workers from employer reprisal, promote unrestricted labor movement (e.g., open work permits) and support workers facing workplace abuse must be explored hand-in-hand with secure status (43, 69).

Likewise, investment in living conditions is crucial to social support as it can change workers’ opportunity to feel more connected to the wider community, access services and supports, and address isolation that can perpetuate abuse (39, 58). Policy reform that would empower workers to make more choices regarding their housing, especially because of its implications for workers’ freedom of movement should be explored (43, 61, 82). Meaningful options for MAWs to escape inadequate housing conditions for example, are warranted (83).

The literature has also identified large gaps in housing inspections and standards for this population (61, 83). Improvements to the oversight of MAWs’ housing could include increasing the volume of unannounced housing inspections, implementing inspections that adhere to rigorous and standardized criteria (58, 83) and stronger collaboration with public health agencies (61). Policies that can prevent MAWs housing from being located near any hazards, such as increased promotion of off-site housing for workers (13, 84) should also be explored.

#### 4.4.2. Promoting mental health and well-being

Several initiatives in mental health promotion and illness prevention have been developed to support MAWs. For instance, Morgaine et al. (85), describes an initiative called the Goodyarn program that was tailored to promote mental health literacy among rural communities, and in this way, work to promote mental health help-seeking and destigmatize mental health challenges among MAWs. In Australia, a government program (Farm-Link) was launched to offer training and the delivery of mental health first aid, with the aims of identifying issues among the farming community (including migrant workers) and connect people to appropriate resources (86). Evaluation of both of these initiatives indicated promising results, yet neither was focused on the specific needs of MAWs, and issues of longevity (86) and a focus on prevention specifically may have limited applicability for those who are already facing mental health challenges. Prior research suggests that migrant mental health is best approached from a multidisciplinary view, in which risk assessments can highlight which interventions are most needed to address MAWs’ mental health needs (73). Thus this may be an important area of research to develop programming that addresses the unique realities of this workforce.

Especially among MAWs with limited social and family networks, faith-based organizations can offer significant support for this group that may contribute to mental wellbeing (41, 78). Indeed, research indicates that migrant workers may engage in spiritual and religious activities such as praying and meditation as coping strategies to overcome acculturative stress. Yet especially for language minority labor groups such as MAWs that are designated as “low-skilled,” opportunities to meaningfully engage with the local community (with language support), fair wages, compensation and support with related paperwork, as well as accessible hotlines with translators and access to mental health professionals are necessary to address the broader challenges that may contribute to poor mental health (60, 73). Likewise, there is a need for social protection and compensation opportunities for work-related psychiatric disorders among MAWs and interventions to reduce job strain that results from workplace insecurity (60, 73). At a broader level, government intervention to guarantee ‘immediate health care upon arrival’ and to increase the accessibility and relevance of health services (43) can have far-reaching implications for mental and physical health alike.

#### 4.4.3. Addressing poor working conditions through occupational health practices and research

As previously outlined, MAWs’ jobs have many hazards and workers face unique challenges around the world due to the structural vulnerabilities they face (42, 49, 61). These broader constraints manifest in poor adherence to health and safety protocols, and limited training about how to enact them (42, 74). Therefore, targeted interventions and programming that enable workers to be active in protecting their health and safety may be one method to address poor working conditions (37). As a first step, the literature suggests identifying training needs of MAWs (2) and multidisciplinary risk assessments (73) toward the systematic development of health and safety programming. The content of training may include requirements to ensure proper PPE adherence, and teaching MAWs proper handling of toxic materials (63, 66). These illness prevention strategies must proactively address literacy and language barriers if they are to effectively be taken up by MAWs (42), and several strategies toward this end have been documented (87), including dedicated government funds to support independent interpretation (60, 61, 69) or health fairs, legal clinics, and outreach clinics for workers (41).

These education efforts require special consideration of migrant workers who are new to working in a host country as they may face increased vulnerabilities (49, 64). Consideration of the rural context in which these health concerns emerge also merit attention (64). Likewise, Wright et al. (37) suggest that worker organizations should play a central role in raising awareness among workers about their rights post-arrival, as a method to promote workers’ ability to self-advocate.

Increased investment in research and data monitoring that can inform occupational illness prevention are also identified as important strategies to keep MAWs safe at work (42, 61, 69). Furthermore, because of the complex interplay of factors that determine occupational health risk, scholars suggest research that more explicitly considers the influence of precarious status (49) and intersecting social identities and processes (e.g., race, class, culture) (40, 52) on workplace health and safety. Through enhanced data monitoring and availability, researchers can also launch longitudinal studies that can inform effective policies for MAWs’ occupational health.

## 5. Conclusion and Implications

Our review identified four key themes related to how current literature may inform our understanding of social support pathways for MAWs. Firstly, under the category of *political, economic and legal factors*, we outlined structural vulnerabilities that undermine MAWs’ ability to access basic services, and assert foundational rights and entitlements. Key factors found across several national contexts included temporary status, deportability, discrimination, limited compensation, and exploitative working conditions. Limits and legal barriers for MAWs to unionize in countries such as Canada and New Zealand present practical challenges. Yet due to converging positional vulnerabilities across all national settings (e.g., deportability), the right to unionize is rarely operationalized, even if enabled by law. With the increased visibility of migrant agricultural workers, and increased urgency to address public health concerns throughout the COVID-19 pandemic, nation-states were called to increase legal protections and regularize the status of this precarious group (14). Yet unfortunately, current research indicates that policies enacted were insufficient, and residency status and precarious employment persisted or even worsened (27, 88). Secondly, *living and working conditions* were discussed across several academic articles, highlighting challenges faced by MAWs in terms of their isolation from the wider community, employer-mediated access to necessary supports, limited and inconsistent access to occupational health, knowledge and resources, and poor day-to-day conditions that jeopardized their health and healthcare seeking. A renewal of commitments to secure migrant agricultural workers’ legal status, strengthen regulatory regimes that would enable them to assert their rights, and strengthening mechanisms for political participation among this group (via labor organizations, housing laws, etc.) could address the root factors that limit workers’ ability to access and navigate a variety of supports. Additional measures that may help to address exploitation include investment in pre-departure and upon arrival training that include connecting to established support networks upon arrival. Policy interventions to address workers’ lived experiences in their countries of origin, such as initiatives to promote greater physical safety, and employment opportunities upon their return, are also warranted, yet have received limited attention (89).

Under *facilitators/barriers to navigating services and supports* we synthesized prior research on MAWs’ language barriers, inaccessibility of services, limited knowledge of supports and entitlements, and other factors determining this groups’ ability to seek timely services. Our last theme, *potential and existing strategies for social support for migrant agricultural workers* captured policy recommendations and existing initiatives that may shed light on strategies to improve support mechanisms for MAWs. Among them, scholars identify: (a) policies that secure the migratory status of MAWs and improve their living conditions, (b) interventions that promote the mental health and wellbeing of this population and; (c) initiatives that address poor working conditions through health and safety protocols and research. These themes emphasize the need for a structural analysis of vulnerabilities faced by migrant agricultural workers, even when addressing downstream or immediate health and social issues. For instance, mental health challenges were mapped in relation to social discrimination, employer-provided housing, poor wages and geographic isolation. While it is encouraging to see beginning investments in MAWs’ mental health and occupational health, limited research documenting interventions addressing other MAWs’ needs were found. Further scholarship, including intervention-based research testing solutions to well-known challenges faced by this group is very much needed. Such projects could explore policy solutions and services (90) to support MAWs facing workplace injury compensation (91), transportation and language barriers (92), human rights abuses (93), job insecurity, wage theft (94), limited spiritual resources, and a lack of community belonging (39, 75, 95).

In short, while much can be learned from existing literature about the broader contextual factors shaping MAWs’ access to and ability to access supports, much less has been written about existing programs and services that can best meet the day-to-day needs of this groups. Interventions and approaches that have been championed with other populations who are underserved, such as language and employment training among immigrants and refugees (96) remote social check-ins/befriendingmodalities with isolated older adults (97) and church-based health promotion strategies with Black diasporas (98) may be transferable, and provider further insight into potential ways forward for service delivery and support for MAWs (99). Despite the paucity of research on such services with MAWs specifically, a strength of the body of literature reviewed in this paper is that it brings focus to the structural domains of vulnerability faced by MAWs and thus invites an analysis of necessary policy interventions that are needed above and beyond ‘band-aid’ solutions to MAWs’ challenges. On the other hand, further research is required to explore what services and supports can best meet the priorities of MAWs, especially when this population remains underserved and underrepresented in program delivery. Mental health promotion initiatives, occupational health education and prevention, along with dedicated outreach and language support are among the more popular initiatives championed. Researchers and service providers alike should further explore other domains of need, such as community connectedness, primary care, legal services, labor representation, and with growing interest in securing the status of this group, further consideration of the role of settlement agencies.

## 6. Limitations

Since we have carried out this review and analysis, additional articles of interest have been published. While we have worked to incorporated some of this scholarship into our paper during later drafts of this publication, our findings should be understood as most relevant to the time-period of focus. Furthermore, issues of increased importance, such as climate change, intricacies of migration policy, country specific labor market dynamics, and comparative research of the impact of public health policies in light of covid-19 were not prominent in prior literature. Consideration of how such issues intersect or inform social support services and initiatives for migrant agricultural workers may be warranted in future research.

## Data availability statement

The original contributions presented in the study are included in the article/Supplementary material, further inquiries can be directed to the corresponding author.

## Author contributions

CSC oversaw the research design and first draft of the paper. ES and CN led secondary searches. All authors contributed to data extraction and thematic analysis of the paper as well as to drafting and final approval of the submitted version.
