# Supplementary material for: Health, social and legal supports for migrant agricultural workers in France, Italy, Spain, Germany, Canada, Australia and New Zealand: a scoping review
Source: Front Public Health. 2023 Oct 5;11:1182816. doi: 10.3389/fpubh.2023.1182816 (PMC10588640; doi:10.3389/fpubh.2023.1182816)
Supplement: Supplementary file 1 [file Data_Sheet_1.PDF]

Appendix A: Search Syntax for Databases

**Keywords >**<br>Concepts >	**Population**<br>Migrant or Domestic Farmworkers<br>Temporary migrants, low-skilled<br>Undocumented/precarious status	**Social Support**<br>Social Support (e.g. psychosocial support, social services, community-based programming)<br>Health promotion/illness prevention, literacy/education, labour union support<br>Individual casework or Treatment or acute intervention	**Region**<br>G7 countries<br>Canada, France, Germany, Italy, Japan, United Kingdom, United States<br>Industrialized country/nation
PubMed	**[Text Word]:**<br>“Farmers” OR “Migrant Worker” OR “Transient” OR Farm worker* OR Guest Worker* OR Migrant Worker* OR Agricultural Worker* OR Seasonal Worker* OR Migrant Laborer OR Migrant Labourer* OR Temporary Migrant OR Foreign Worker	**[Text Word]:**<br>“Social Environment” OR “Social Support” OR “Psychosocial Support Systems” OR “Social Work”OR “Social Planning” OR “Community Integration” OR “Community Outreach” OR “Health Services Accessibility” OR “Communication Barriers” OR “Health Services” OR “Community Health Services” OR “Community Mental Health Services” OR “Occupational Health” OR “Occupational Illness” OR “Occupational Health Services” OR “Occupational Health Nursing” OR “Community Health Nursing” OR “Community Mental Health Nursing” OR “Parish Nursing” OR “Social Workers” OR “Health Promotion” OR “Health Promoter*” OR “Community Health Workers” OR “Community Psychiatry”<br>OR “Patient Advocacy”	**[Text Word]:**<br>“Canada” OR “France” OR “Germany” OR “Italy” OR “ Japan” OR “ United Kingdom” OR “United States” OR “Australia” OR “New Zealand” OR “Spain” OR “Developed Countries”
SCOPUS	**TITLE-ABS-KEY:**<br>Farmworker* OR Farmer OR "Guest Work*" OR "Migrant Work*" OR "Agricultural Work*" OR "Seasonal Work*" OR "Migrant Labor" OR "Migrant Laborer*" OR "Migrant Labour*"	**TITLE-ABS-KEY:**<br>(Community W/2 Outreach) OR (Community W/2 Empowerment) OR (Community W/2 Health) OR (Community W/2 Services) OR (Community W/2 Nursing) OR (Community W/2 Support) OR (Social W/2 Support) OR (Psychosocial W/2 Support) OR (Support W/2 Group*)<br>**OR**<br>(Service* W/2 Access*) OR (Access* W/2 Barrier*) OR (Communication W/2 Barrier*) OR (Access* W/2 Information) OR (Access W/2 Justice) OR (Community W/2 Advoc*) OR (Patient W/2 Advoc*) OR (Legal W/2 Advoc*) OR (Health W/2 Promotion) OR (Health W/2 Safety)<br>**OR**<br>"Parish Nursing" OR "Social Worker*" OR "Health Promoter*" OR "Occupational Health" OR "Occupational Illness" OR "Social Support"	**TITLE-ABS-KEY:**<br>Canada OR France OR Germany OR Italy OR Japan OR England OR "United Kingdom" OR "Great Britain" OR "United States" OR "G7 countries" OR Australia OR Spain OR "New Zealand" OR "Industrialized Nation*" OR "Developed Countr*"
ProQuest (PsycInfo & Sociology	**Anywhere except full text (NOFT)**	**Anywhere except full text (NOFT)**	**Anywhere except full text (NOFT)**

Collection)	Farmworker* OR Farmer OR "Guest Work*" OR "Migrant Work*" OR "Agricultural Work*" OR "Seasonal Work*" OR "Migrant Labor" OR "Migrant Laborer*" OR "Migrant Labour*"	"Community Mental Health Nursing" OR "Community Health Centers" OR "Community Networks" OR "Community Health Nursing" OR "Parish Nursing" OR "Social Worker*" OR "Health Promoter*" OR "Occupational Health" OR "Occupational Illness" OR "Social Support" OR "Social Workers" OR "Social Environment" OR "Health Promotion" OR "Mental Health Promotion" OR "Occupational Health Services" OR "Occupational Health Nursing"<br>**OR**<br>"social support*" OR "social support model" OR "social support intervention" OR "support system" OR "community outreach" OR "community empowerment" OR "occupational health" OR "occupational illness" OR "illness prevention" OR "legal advocacy" OR "access to justice" OR "service access" OR "improving access" OR "support barriers" OR "community outreach" OR "Community Empowerment" OR "Community Support" OR "patient Advocacy" OR "Health Services Accessibility" OR "Access to Information" OR "Communication Barriers" OR "Support Groups"	Canada OR France OR Germany OR Italy OR Japan OR England OR "United Kingdom" OR "Great Britain" OR "United States" OR "G7 countries" OR Australia OR Spain OR "New Zealand" OR "Industrialized Nation*" OR "Developed Countr*"
Web of Science	**TS= Topic OR TI= Title**<br>Farmworker* OR Farmer OR "Guest Work*" OR "Migrant Work*" OR "Agricultural Work*" OR "Seasonal Work*" OR "Migrant Labor" OR "Migrant Laborer*" OR "Migrant Labour*"	**TS= Topic OR TI= Title**<br>"social support*" OR "social support model" OR "social support intervention" OR "support system" OR "community outreach" OR "community empowerment" OR "occupational health" OR "occupational illness" OR "illness prevention" OR "legal advocacy" OR "access to justice" OR "service access" OR "improving access" OR "support barriers" OR "community outreach" OR "Community Empowerment" OR "Community Support" OR “patient Advocacy” OR “Health Services Accessibility” OR “Access to Information” OR “Communication Barriers” OR “Support Groups”<br>OR<br>"Community Mental Health Nursing" OR "Community Health Centers" OR "Community Networks" OR "Community Health Nursing" OR “Parish Nursing” OR “Social Worker*” OR “Health Promoter*” OR “Occupational Health” OR “Occupational Illness” OR “Social Support” OR “Social Workers” OR “Social Environment” OR “Health Promotion” OR “Mental Health Promotion” OR “Occupational Health Services” OR “Occupational Health Nursing”	**TS= Topic OR TI= Title**<br>Canada OR France OR Germany OR Italy OR Japan OR England OR ”United Kingdom" OR "Great Britain" OR "United States" OR "G7 countries" OR Australia OR Spain OR “New Zealand" OR “Industrialized Nation*" OR “Developed Countr*”

Appendix B: Search terms for additional select European countries search and secondary Google Scholar search

Population/Setting
Temporary migrant workers<br>Migrant agricultural workers<br>Migrant farmworkers<br>Agriculture
**Concept/phenomenon/intervention**
Social support<br>Social protection<br>Support intervention<br>Health services<br>Social services<br>Settlement services<br>Legal services<br>Community belonging
**Geographic focus***
Germany<br>France<br>Spain<br>Italy<br>*applied after conducting a general search to address limited scholarship in these contexts

Appendix C: List of articles included in scoping review analysis

1) Achard, Pauline, et al. "Medico-administrative data combined with agricultural practices data to retrospectively estimate pesticide use by agricultural workers." Journal of Exposure Science & Environmental Epidemiology (2020) 30(4): 743-755.

2) Akbar M. 2022 Temporariness and the production of policy categories in Canada. J Ethn Migr Stud (2022) 48(16): 3929-3946.

3) Anderson, G., & Kenner, L. (2019). Enhancing the effectiveness of minimum employment standards in New Zealand. The Economic and Labour Relations Review 30(3):345-365.

4) Andersson E, Lundqvist P. Gendered agricultural space and safety: Towards embodied, situated knowledge. Journal of agromedicine (2014) 19(3):303-315.

5) Bagagiolo G, Vigoroso L, Caffaro F, Micheletti Cremasco M, Cavallo E. Conveying safety messages on agricultural machinery: the comprehension of safety pictorials in a group of migrant farmworkers in Italy. International journal of environmental research and public health (2019) 16(21):4180.

6) Barnes N. Is health a labour, citizenship or human right? Mexican seasonal agricultural workers in Leamington, Canada. Global Public Health 2013 8(6):654-669.

7) Barnetson B. No right to be safe: Justifying the exclusion of Alberta farm workers from health and safety legislations. Socialist Studies/Études Socialistes (2012).

8) Basok T, George G. We are part of this place, but I do not think I belong: Temporariness, social inclusion and belonging among migrant farmworkers in southwestern Ontario. Int Migr (2021) 59: 99-112.

9) Basok T, López-Sala A. Rights and restrictions: Temporary agricultural migrants and trade unions' activism in Canada and Spain. J Int Migr Integr (2016) 17(4): 1271-1287.

10) Benach J, Muntaner C, Chung H, Benavides FG. Immigration, employment relations, and health: Developing a research agenda. Am J Ind Med (2010) 53:338-343.

11) Besser T, Just C, Mann S. Agricultural structure and farmers' interconnections with rural communities. Int J Soc Econ (2017) 44(3):362-376.

12) Blackman IR. Factors influencing australian agricultural workers' self-efficacy using chemicals in the workplace. Workplace Health Saf (2012) 60(11):489-496.

13) Blaker L. Identifying the health needs of seasonal workers from the Pacific. N Z Nurs J (2019) 25(8): 36-38.

14) Boccagni P. Caring about migrant care workers: From private obligations to transnational social welfare? Crit Soc Policy (2014) 34(2): 221-240.

15) Brickenstein C. Social protection of foreign seasonal workers: from state to best practice. Comp Migr Stud (2015) 3(1): 1-18.

16) Caffaro F, Cremasco MM, Bagagiolo G, Vigoroso L, Cavallo E. Effectiveness of occupational safety and health training for migrant farmworkers: a scoping review. Public health (2018) 160:10-17.

17) Callejón-Ferre ÁJ, Montoya-García ME, Pérez-Alonso J, Rojas-Sola JI. The psychosocial risks of farm workers in south-east Spain. Safety science (2015) 78:77-90.

18) Carney JG, Gushulak BD. A review of research on health outcomes for workers, home and host communities of population mobility associated with extractive industries. J Immigr Minor Health (2016) 18(3): 673-686.

19) Caxaj CS, Cohen A. Emerging best practices for supporting temporary migrant farmworkers in Western Canada. Health Soc Care Community (2021) 29(1):250-258.

20) Caxaj CS, Cohen A. Relentless border walls: Challenges of providing services and supports to migrant agricultural workers in British Columbia. Can Ethn Stud (2021) 53(2):41-67.

21) Caxaj CS, Cohen A, Colindres C. More of the same? Migrant agricultural workers' health, safety, and legal rights in the COVID-19 context. J Agric Food Syst Community Dev (2022) 11(3):1-18.

22) Caxaj CS, Diaz L. Migrant workers' (non)belonging in rural British Columbia, Canada: storied experiences of marginal living. Int J Migr Health Soc Care (2018) 14(2):208-220.

23) Cole DC, McLaughlin JE, Hennebry JL, Tew MA. Precarious patients: Health professionals' perspectives on providing care to Mexican and Jamaican migrants in Canada's Seasonal Agricultural Worker Program. Rural Remote Health (2019) 19(4):5313.

24) Colindres C, Cohen A, Caxaj CS. Migrant agricultural workers' health, safety and access to protections: A descriptive survey identifying structural gaps and vulnerabilities in the interior of British Columbia, Canada. Int J Environ Res Public Health (2021) 18(7): 3696.

25) Corrado A, Palumbo L. "Essential farmworkers and the pandemic crisis: Migrant labour conditions, and legal and political responses in Italy and Spain". In: Migration Pandemics: Spaces of Solidarity and Spaces of Exception. (2022) 145-166.

26) Dines N, Rigo E. “Postcolonial citizenship between representation, borders and the ‘refugeeization’ of workforce: Critical reflections on migrant agricultural labour in the Italian Mezzogiorno” In: Ponzanesi G, Colpani G, editor. Rowman and Littlefield. London (2015). P. 155-172.

27) Gabriel C, Macdonald L. Citizenship at the Margins: The Canadian Seasonal Agricultural Worker Program and Civil Society Advocacy 1. In North American Integration (2013) 115-135.

28) Gabriel C, Macdonald L. After the international organization for migration: recruitment of Guatemalan temporary agricultural workers to Canada. J Ethn Migr Stud (2018) 44(1): 1706-1724.

29) Government of British Columbia. Quarantine for foreign agricultural, food, and seafood workers who are not fully vaccinated (2022) https://www2.gov.bc.ca/gov/content/industry/agriculture-seafood/covid-19-response/temporary-foreign-farmworkers [Accessed January 30, 2023].

30) Government of Canada. Government of Canada invests in measures to boost protections for temporary foreign workers and address COVID-19 outbreaks on farms. (2020). https://www.canada.ca/en/employment-social-development/news/2020/07/government-of-canada-invests-in-measures-to-boost-protections-for-temporary-foreign-workers-and-address-covid-19-outbreaks-on-farms.html [Accessed January 30, 2023].

31) Hanley J, Paul L, Ravinthiran J, Malhaire L, Mosseau N. Protecting the rights of migrant farmworkers in Quebec: To what extent can unionization overcome the effects of precarious immigration status? J Rural Community Dev (2020) 15(2).

32) Hedberg C. Entwined ruralities: Seasonality, simultaneity and precarity among transnational migrant workers in the wild berry industry. J Rural Stud (2021) 88:510-517.

33) Hennebry JL, Preibisch K. A model for managed migration? Re-examining best practices in Canada’s seasonal agricultural worker program. Int Migr (2012) 50:e19-e40.

34) Hennebry J, McLaughlin J, Preibisch K. Out of the loop: (In)access to health care for migrant workers in Canada. J Int Migr Integr (2016) 17(2): 521-538.

35) Isaac M, Elrick J. How COVID-19 may alleviate the multiple marginalization of racialized migrant workers. Ethn Racial Stud (2020) 44(5):851-863.

36) Landry V, Semsar-Kazerooni K, Tjong J, Alj A, Darnley A, Lipp R, Guberman, GI. The systemized exploitation of temporary migrant agricultural workers in Canada: Exacerbation

of health vulnerabilities during the COVID-19 pandemic and recommendations for the future. J Migr Health (2021) 3:100035.

37) Liem A, Renzaho AM, Hannam K, Lam AI, Hall BJ. Acculturative stress and coping among migrant workers: A global mixed-methods systematic review. Appl Psychol Health Well Being (2021) 13(3): 491-517.

38) Liem A, Wang C, Wariyanti Y, Latkin CA, Hall BJ. The neglected health of international migrant workers in the COVID-19 epidemic. Lancet Psychiatry (2020) 7(4): e20.

39) López-Jacob MJ, Safont EC, García AM, Garí A, Agudelo-Suárez A, Gil A, Benavides FG. Participation and influence of migrant workers on working conditions: a qualitative approach. New solutions: a journal of environmental and occupational health policy (2010) 20(2):225-238.

40) Lozanski K, Baumgartner K. Local gastronomy, transnational labour: farm-to-table tourism and migrant agricultural workers in Niagara-on-the-Lake, Canada. Tour Geogr (2020) 24(2): 1-23.

41) Lundqvist P, Day L. The 2008 Saskatoon Declaration for the Health, Safety, and Living Conditions of Migrant Workers in Agriculture: Proposed at the Sixth International Symposium: Public Health and the Agricultural-Rural Ecosystem, Saskatoon, Canada (2008).

42) Kilkey M, Urzi D. Social reproduction in Sicily's agricultural sector: migration status and context of reception. J Ethn Migr Stud (2017) 43(15): 2573-2590.

43) Kosny A, Allen AR. Falling through the cracks? An analysis of health and safety resources for migrant workers in Australia. Int J Migr Health Soc Care (2016) 12(2):99-108.

44) Macfarlane E, Chapman A, Benke G, Meaklim J, Sim M, McNeil J. Training and other predictors of personal protective equipment use in Australian grain farmers using pesticides. Occup Environ Med (2008) 65(2):141-146.

45) Mammone T, Metruccio F, Vida P, Moretto A. The Italian system of data reporting in agriculture occupational health: A critical appraisal. J Public Health (2007) 15(4):301-313.

46) Morgaine K, Thompson L, Jahnke K, Llewellyn R. GoodYarn: building mental health literacy in New Zealand’s rural workforce. J Public Ment Health (2017) 16(4): 180-190.

47) Marsh G, Lewis V, Macmillan J, Gruszin S. Workplace wellness: industry associations are well placed and some are ready to take a more active role in workplace health. BMC Health Serv Res (2018) 18:1-5

48) McBain-Rigg KE, Franklin RC, King JC, Lower T. Influencing safety in Australian agriculture and fisheries. Journal of agromedicine (2017) 22(4):347-357.

49) Meardi G, Martín A, Riera ML. Constructing uncertainty: Unions and migrant labour in construction in Spain and the UK. J Ind Relat (2012) 54(1):5-21.

50) Migrant Worker Health Expert Working Group. Recommendations for overcoming health challenges faced by migrant agricultural workers during COVID-19 Virus Pandemic (2020). https://crhesi.uwo.ca/wp-content/uploads/2020/05/May-25-HC-ChallengesGaps-Recommendations.pdf [Accessed February 16, 2023].

51) Ministero del Lavoro e delle Politiche Sociali. Italy: Combating the exploitation of migrant workers in agriculture (2022). https://ec.europa.eu/migrant-integration/news/italy-combating-exploitation-migrant-workers-agriculture_en [Accessed January 30, 2023].

52) Molinero-Gerbeau Y, López-Sala A, Șerban M. On the social sustainability of industrial agriculture dependent on migrant workers. Romanian workers in Spain's seasonal agriculture. Sustainability (2021) 13(3):1062.

53) Narushima M, McLaughlin J, Barrett-Greene J. Needs, risks, and context in sexual health among temporary foreign migrant farmworkers in Canada: A pilot study with Mexican and Caribbean workers. J Immigr Minor Health (2016) 18(2):374-381.

54) Narushima M, Sanchez AL. Employers paradoxical views about temporary foreign migrant workers health: a qualitative study in rural farms in Southern Ontario. International Journal for Equity in Health (2014) 13(1):1-12.

55) Neef A. Legal and social protection for migrant farm workers: Lessons from COVID-19. Agric Human Values (2020) 37:641-642.

56) Nguyen HT, Quandt SA, Grzywacz JG, Chen H, Galván L, Kitner-Triolo MH, Arcury TA. Stress and cognitive function in Latino farmworkers. American journal of industrial medicine (2012) 55(8):707-713.

57) Ollé-Espluga L, Vergara-Duarte M, Belvis F, Menéndez-Fuster M, Jódar P, Benach J. What is the impact on occupational health and safety when workers know they have safety representatives? Saf Sci (2015) 74:55-58.

58) Peach HG. Migrant farmworkers and health. Rural Remote Health (2013) 13(2): 1-3.

59) Perceval M, Fuller J, Holley AM. Farm-link: Improving the mental health and well-being of people who live and work on NSW farms. Int J Ment Health (2011) 40(2):88-110.

60) Perry JA. Images of work, images of defiance: engaging migrant farm worker voice through community-based arts. In Critical Adult Education in Food Movements. Springer Nature Switzerland (2022) 107-120.

61) Porru S, Elmetti S, Arici C. Psychosocial risk among migrant workers: what we can learn from literature and field experiences. Med Lav (2014) 105(2):109-129.

62) Preibisch K, Otero G. Does citizenship status matter in Canadian agriculture? Workplace health and safety for migrant and immigrant laborers. Rural Sociol (2014) 79(2): 174-199.

63) Robillard C, McLaughlin J, Cole DC, Vasilevska B, Gendron R. "Caught in the same webs"—service providers' insights on gender-based and structural violence among female temporary foreign Workers in Canada. Journal of International Migration and Integration (2018) 19:583-606.

64) Ronda-Pérez E, Gosslin A, Martínez JM, Reid A. Injury vulnerability in Spain. Examination of risk among migrant and native workers. Saf Sci (2019) 115:36-41.

65) Russo R. Collective struggles: A comparative analysis of unionizing temporary foreign farm workers in the United States and Canada. Houst J Int Law (2018) 41(5).

66) Salami B, Meherali S, Salami A. The health of temporary foreign workers in Canada: A scoping review. Can J Public Health (2016) 106(8):e546-54.

67) Smith AA. Racialized in justice: The legal and extra-legal struggles of migrant agricultural workers in Canada. Windsor Yrbk Acc Jus (2013) 31(2):15.

68) Sousa E, Agudelo-Suárez A, Benavides FG, Schenker M, García AM, Benach J, Delclos C, López-Jacob MJ, Ruiz-Frutos C, Ronda-Pérez E, Porthé V. Immigration, work and health in Spain: The influence of legal status and employment contract on reported health indicators. Int J Public Health (2010) 55(5):443-451.

69) Stevano S, Ali R, Jamieson M. Essential for what? A global social reproduction view on the re-organisation of work during the COVID-19 pandemic. Rev Can Etudes Dev (2021) 42(1-2):178-199

70) Tagliacozzo S, Pisacane L, Kilkey M. The interplay between structural and systemic vulnerability during the COVID-19 pandemic: Migrant agricultural workers in informal settlements in Southern Italy. J Ethn Migr Stud (2021) 47(9):1903-1921 .

71) Taylor A, Foster J. Migrant workers and the problem of social cohesion in Canada. Journal of International Migration and Integration (2015) 16:153-172.

72) Toh S, Quinlan M. Safeguarding the global contingent workforce? Guestworkers in Australia. Int J Manpow (2009) 30(5):453-471.

73) Triandafyllidou A. Migration and Pandemics: Spaces of Solidarity and Spaces of Exception. Springer Nature, Canada (2022).

74) Tutt D, Pink S, Dainty ARJ, Gibb A. In the air and below the horizon: migrant workers in UK construction and the practice-based nature of learning and communicating OHS. Constr Manag Econ (2013) 31(6):515-527.

75) US Department of Agriculture. Farm labor stabilization and protection pilot grant program (2022). https://www.fsa.usda.gov/farmworkers/index [Accessed January 30, 2023].

76) Vahabi M, Wong JP. Caught between a rock and a hard place: mental health of migrant live-in caregivers in Canada. BMC Public Health (2017) 17(1):498.

77) Van Haren I, Masferrer C. Mexican migration to Canada: Temporary worker programs, visa imposition, and NAFTA shape flows. SocArXiv (2022).

78) Vigoroso L, Caffaro F, Micheletti Cremasco M, Bagagiolo G, Cavallo E. Comprehension of safety pictograms affixed to agricultural machinery among Pakistani migrant farmworkers in Italy. J Agromedicine (2020) 25(3):265-278.

79) Viveros-Guzmán A, Gertler M. Latino farmworkers in Saskatchewan: Language barriers and health and safety. J Agromedicine (2015) 20(3):341-348.

80) Vosko LF, Basok T, Spring C, Candiz G, George G. Understanding migrant farmworkers' health and well-being during the global COVID-19 pandemic in Canada: Toward a transnational conceptualization of employment strain. Int J Environ Res Public Health (2022) 19(14):8574.

81) Weiler AM, McLaughlin J. Listening to migrant workers: should Canada's Seasonal Agricultural Worker Program be abolished?. Dialectical Anthropology (2019) 43(4):381-888.

82) Wong JPH, Kwong M, Poon L, Vahabi M, Sutdhibhasilp N, Narushima M. Transnational contexts and local embeddedness of HIV/STI vulnerabilities among Thai and Filipino agricultural temporary foreign workers in Canada. Cult Health Sex (2020) 23(6):723-739.

83) Wright CF, Groutsis D, Kaabel A. Regulating migrant worker temporariness in Australia: the role of immigration, employment and post-arrival support policies. J Ethn Migr Stud (2022) 48(16): 3947-3964.

84) Zoeteweij-Turhan MH. The seasonal workers directive: '… but some are more equal than others. Eur Labour Law J (2017) 8(1): 28-44.
